# Evaluation of the WHO 2010 Grading and AJCC/UICC Staging Systems in Prognostic Behavior of Intestinal Neuroendocrine Tumors

**DOI:** 10.1371/journal.pone.0061538

**Published:** 2013-04-19

**Authors:** Paula B. Araujo, Sonia Cheng, Ozgur Mete, Stefano Serra, Emilie Morin, Sylvia L. Asa, Shereen Ezzat

**Affiliations:** 1 Department of Medicine, University Health Network, Toronto, Ontario, Canada; 2 Department of Pathology, University Health Network, Toronto, Ontario, Canada; The Chinese University of Hong Kong, Hong Kong

## Abstract

**Background:**

The increasing incidence and heterogeneous behavior of intestinal neuroendocrine tumors (iNETs) pose a clinicopathological challenge. Our goal was to decribe the prognostic value of the new WHO 2010 grading and the AJCC/UICC TNM staging systems for iNETs. Moreover, outcomes of patients treated with somatostatin analogs were assessed.

**Methods:**

We collected epidemiological and clinicopathological data from 93 patients with histologically proven iNETs including progression and survival outcomes. The WHO 2010 grading and the AJCC/UICC TNM staging systems were applied for all cases. RECIST criteria were used to define progression. Kaplan-Meier analyses for progression free survival (PFS) and overall survival (OS) were performed.

**Results:**

Mean follow-up was 58.6 months (4–213 months). WHO 2010 grading yielded PFS and disease-specific OS of 125.0 and 165.8 months for grade 1 (G1), 100.0 and 144.2 months for G2 and 15.0 and 15.8 months for G3 tumors (p = 0.004 and p = 0.001). Using AJCC staging, patients with stage I and II tumors had no progression and no deaths. Stage III and IV patients demonstrated PFS of 138.4 and 84.7 months (p = 0.003) and disease-specific OS of 210.0 and 112.8 months (p = 0.017). AJCC staging also provided informative PFS (91.2 vs. 50.0 months, p = 0.004) and OS (112.3 vs. 80.0 months, p = 0.005) measures with somatostatin analog use in stage IV patients.

**Conclusion:**

Our findings underscore the complementarity of WHO 2010 and AJCC classifications in providing better estimates of iNETS disease outcomes and extend the evidence for somatostatin analog benefit in patients with metastatic disease.

## Introduction

Neuroendocrine tumors (NETs), also known as “carcinoid” tumors, are slowly growing neoplasms that were previously considered to be largely benign, however, retrospective data suggested that all NETs have malignant potential [1]. Gastrointestinal (GI) tract NETs account for 67% of NETs; the small bowel is the most frequent primary site (42%) within this group [2]. Further, NETs account for 37% of all small bowel cancers [3].

The incidence of NETs has increased from 1.09 to 5.25/100,000 per year [4], associated with a rise in the 5 year survival rate from 59% in the 1970s and 1980s to 67% in the 1990s [2]. The incidence is slightly higher among males [4] and the median age of presentation is 64 years; patients with appendiceal tumors are younger at diagnosis, with a median age of 47 years [5].

Intestinal NETs (iNETs) arising in the small bowel, appendix and large bowel are typically discovered incidentally during surgery or imaging for unexplained symptoms [6]–[8]. When symptoms occur they tend to be nonspecific, often vague abdominal pain; carcinoid syndrome appears in just 20–30% of the patients, who almost invariably have metastases [9]. Surgery remains the only potentially curative therapy for patients with localized disease [5], [9]. Palliative resection, liver transplantation, peptide receptor radiotherapy (PRRT), and local ablative/loco-regional techniques including radiofrequency ablation (RFA), hepatic embolization, and chemoembolization, are reserved for patients with metastatic disease [9]–[11].

Medical therapy is limited and not curative, having two major goals: anti-secretory and anti-proliferative effects. Somatostatin analogs (SA) are the most commonly used drugs to control hormone hypersecretion [5], [10] potentially with added anti-proliferative actions [12] as demonstrated by the PROMID study [13]. Other therapies used alone or in combination with SA include: interferon [14], chemotherapy [15] and the molecular targeted therapies mTOR inhibitors and VEGF inhibitors [16], [17].

Given the increasing incidence and wide biological spectrum of NETs, prognostic factors that predict long-term outcomes and can guide therapy are needed. The WHO 2010 classification of gastroenteropancreatic (GEP) NETs introduced a three tier system that integrates the mitotic count (MC) and Ki-67 (MIB-1) labeling index with differentiation of these neoplasms [1]. However, this classification has limited ability to predict the biological aggressiveness of NETs since low grade NETs can also metastasize.

The limitations of this classification led to efforts to create a unified system based on TNM staging [18]. Therefore, TNM staging systems were proposed by the European Neuroendocrine Tumor Society (ENETS) [9] and by the American Joint Committee on Cancer/Union Internationale Contre le Cancer (AJCC/UICC) [5], giving rise to two parallel systems. The most recent 7^th^ AJCC/UICC TNM staging system introduced a site-specific and grade-dependent staging model for GEP-NETs [19]. Although the WHO grading and AJCC/UICC staging systems have been introduced recently, the prognostic impact of these classifications has not been widely validated for iNETs. Therefore, we aimed to evaluate the prognostic impact of these systems retrospectively in our institutional cohort of iNETs. We also applied these classifications in assessing the long-term follow-up of patients who underwent biotherapy with somatostatin analogs.

## Patients and Methods

### Patients

This retrospective assessment collected clinical data and treatment outcomes from the medical charts of 93 consecutive patients with histologically confirmed iNETs diagnosed from 1994 to 2011 at the University Health Network (UHN), a tertiary referral center for the management of NETs in Toronto, Ontario, Canada. The study was approved by the UHN Research Ethics Board. Written consent was provided for patient information to be stored and used for research purposes.

Patient charts were reviewed to collect the following data: demographic features (age and gender), medical history and comorbidities, biochemical data [urinary 5-HIAA excretion and serum chromogranin A (CgA)], clinical features of carcinoid syndrome, diagnostic imaging investigations (octreoscanning, computed tomography, MRI), primary tumor location, histopathological features (size and site of primary tumor, mitotic count (MC) and Ki-67 index, lymph node involvement, vascular invasion, depth of invasion and immunohistochemical staining), presence of metastasis, treatment modalities and survival outcomes.

### Tumor grading and clinical staging

Grading was performed following the WHO 2010 classification [1], [19] according to their proliferative rates as follows: G1: <2 mitoses/10 HPF and <3% Ki-67 labeling index, G2: 2–20 mitoses/10 HPF or 3–20% Ki-67 labeling index, G3: >20 mitoses/10 HPF or >20% Ki-67 labeling index. In instances where the MC and the Ki-67 labeling index provided conflicting information, the higher value was adopted for grading purposes.

For staging, we used the TNM classification for neuroendocrine tumors from the 7^th^ Edition of the AJCC/UICC [5]: stages IIA–IIB were grouped in stage II and stages IIIA–IIIB were grouped in stage III. Combined information from CT/MRI imaging and/or surgical pathology report was used to perform tumor staging. Other imaging modalities such as nuclear bone scans and octreoscan were used to determine extent of metastatic disease.

### Assessment of clinical outcomes

*Overall survival* (OS) was defined as the number of months from the date of diagnosis, defined by the first diagnostic imaging study, to the date of the last follow-up visit or time of death while *disease-specific OS* was measured from date of diagnosis to date of the last follow-up visit or time of death attributed to iNET. Deaths classified as not being related to iNETs included 6 cases as follows: myocardial infarction, uterine sarcoma, rectal carcinoma, lung adenocarcinoma, prostate carcinoma, and Merkel cell carcinoma.

*Progression free survival* (PFS) was defined as the number of months from the date of first therapeutic intervention (therapeutic surgery, palliative surgery or locoregional procedure, SA treatment) to the first documentation of disease recurrence, progression, or death by any cause. Disease progression or recurrence status was determined on the basis of objective imaging studies according to RECIST criteria [20].

### Statistical analyses

All variables were reported according to their distribution by means, medians, standard deviations (SD), variance, minimum, maximum or range and their frequencies as proportions (%). We used t-tests to compare means or Mann-Whitney U test according to variable distribution. We performed analysis of survival with Kaplan-Meier curves and comparisons between factors and strata when necessary. For comparisons in survival analysis we used generalized Wilcoxon test between factors. Significant variables were also tested in a multivariate analysis using Cox proportional hazards regression model. Statistical significance was considered reached when p-values were below 0.05.

## Results

### Patient characteristics

The clinicopathologic data of the patient cohort are summarized in Table 1. Mean follow-up was 58.6 months (4–213 months) and mean age at diagnosis was 56.6 years (SD±15.0) with an equal gender distribution (50.5% male). The most common presentation at diagnosis was abdominal pain (33.3%), although 34.4% of patients developed carcinoid syndrome at some point during follow-up. Preoperative imaging studies (CT and/or MRI) revealed that the most common initial radiographic findings were the presence of a small bowel lesion in 33.3% and a mesenteric mass associated with a bowel lesion in 22.6%. Distant metastatic disease was present in 43.0% (n = 40) of patients and the liver, alone or in combination with other sites, was the most frequently involved organ. Octreoscan studies were available for 70.9% (n = 66) of patients showing avidity in 46.9% of this group. Serum CgA and urinary 5-HIAA levels were available for 71 (65.6%) and 76 (81.7%) patients respectively and were increased in 35.2% and 42.1% of tested patients, respectively.

**Table 1:** Clinical and pathological features.

	All patients, n (%)
Age (years)	56.6±15.0*
Male	47 (50.5)
Diagnosis presentation	
Upper endoscopy	2 (2.2)
Colonoscopy	7 (7.5)
Appendicitis	12 (12.9)
Incidental mass	22 (23.7)
Abdominal pain	31 (33.3)
Carcinoid symptoms	19 (20.4)
Family history of cancer	43 (46.2)
History of other cancers	25 (26.9)
Carcinoid syndrome	32 (34.4)
Biochemical markers	
CgA total tested/increased	71/25 (65.6/26.9)
5-HIAA total tested/increased	76/32 (81.7/34.4)
MRI/CT Findings	
Duodenum	3 (3.2)
Small bowel	31 (33.3)
Appendix	10 (10.8)
Cecum or right colon	3 (3.2)
Proximal transverse colon	1 (1.1)
Mesenteric mass	21 (22.6)
Unknown	24 (25.8)
MRI/CT Primary site size (cm)	2.7±1.8*
Site of metastases	
Regional LN	17 (18.3)
Liver	37 (39.8)
Lung	1 (1.1)
Liver and lung	1 (1.1)
Liver and bone	1 (1.1)
MRI/CT Liver metastases size (cm)	3.8±2.4*
Octreoscan avidity	
Liver	12 (12.9)
Abdomen	16 (17.2)
Other	3 (3.2)
Negative	35 (37.6)
NA	27 (29.0)
Pathology size (cm)	2.4±1.5*
Pathology primary site	
Duodenum	4 (4.3)
Jejunum	24 (25.8)
Ileum	42 (45.2)
Appendix	17 (18.3)
Cecum	1 (1.1)
Proximal transverse colon	1 (1.1)
Unknown	4 (4.3)
Vascular Invasion	45 (48.4)
LN involvement	71 (76.3)

Abbreviations: CgA, chromogranin A; 5-HIAA, 5-hydroxyindolacetic acid; LN, lymph node; NA, not available.

* Plus–minus values are means ± SD.

There were 17 primary appendiceal tumors and 12 of these were associated with appendicitis. The most common anatomic primary site was the ileum (45.2%) followed by the jejunum (25.8%), with multifocal bowel disease in 12 cases (12.9%). Immunohistochemistry (IHC), available for 74 cases, showed that CgA was positive in 81.0% and serotonin in 56.7% of cases.

### Therapeutic interventions

Overall, 92.5% (n = 86) of patients underwent tumor resection with intestinal resection in 88.2% (n = 82) and mesenteric lymph node resection in 80.6% (n = 75). Resection of liver metastasis was carried out in 26.9% (n = 25) of patients. Loco-regional therapies included liver embolization and RFA in 12.9% (n = 12) and 5.4% (n = 5) of patients, respectively. Systemic therapy with SA was given to 44.1% (n = 41) of patients in the form of octreotide LAR. Chemotherapy was administered in 6 patients (6.5%) and the cytotoxic drugs employed were platinum compounds (6 patients), etoposide (5 patients), and 5-fluorouracil (2 patients). Palliative radiotherapy was applied in 9.7% (n = 9) of patients.

### WHO Grading and AJCC/UICC Staging

WHO 2010 grading was possible for 77 patients with a distribution of 37, 36 and 4 patients with grades 1, 2 and 3 tumors respectively. AJCC/UICC staging classified 8, 8, 37 and 40 patients into stages I, II, III and IV, respectively. Analyses for both classifications are summarized in Table 2.

**Table 2:** WHO grading and AJCC staging analyses.

	WHO grading					AJCC staging				
	G1	G2	G3	NA	p value	I	II	III	IV	p value
	N (%)	N (%)	N (%)	N (%)		N (%)	N (%)	N (%)	N (%)	
Distribution	37 (39.8)	36 (38.7)	4 (4.3)	16 (17.2)		8 (8.6)	8 (8.6)	37 (39.8)	40 (43.0)	
Dx Presentation										
Upper endoscopy	1 (2.7)	0 (0)	0 (0)	1 (6.2)	NS	0 (0)	1 (12.5)	1 (2.7)	0 (0)	NS
Colonoscopy	2 (5.4)	4 (11.1)	1 (25.0)	0 (0)	NS	1 (12.5)	1 (12.5)	4 (10.8)	1 (2.5)	NS
Appendicitis	9 (24.3)	0 (0)	0 (0)	3 (18.0)	0.009	7 (87.5)	3 (37.5)	2 (5.4)	0 (0)	<0.001
Incidental mass	7 (18.9)	11 (30.5)	0 (0)	4 (25.0)	NS	0 (0)	1 (12.5)	11 (29.7)	10 (25.0)	NS
Abdominal pain	9 (24.3)	12 (33.3)	3 (75.0)	7 (43.8)	NS	0 (0)	1 (12.5)	16 (43.2)	14 (35.0)	NS
Carcinoid symptoms	9 (24.3)	9 (25.0)	0 (0)	1 (6.2)	NS	0 (0)	1 (12.5)	3 (8.1)	15 (37.5)	<0.001
Pathology primary site										
Duodenum	2 (5.4)	0 (0)	1 (25.0)	1 (6.2)	0.041	0 (0)	1 (12.5)	2 (5.4)	1 (2.5)	NS
Jejunum	8 (21.6)	12 (33.3)	0 (0)	4 (25.0)	NS	0 (0)	2 (25.0)	10 (27.0)	12 (30.0)	NS
Ileum	14 (37.8)	21 (58.3)	1 (25,0)	6 (37.5)	NS	1 (12.5)	1 (12.5)	21 (56.8)	19 (47.5)	0.001
Appendix	11 (29.7)	1 (2.8)	1 (25.0)	4 (25.0)	0.041	7 (87.5)	4 (50.0)	4 (10.8)	2 (5.0)	0.001
Cecum	0 (0)	1 (2.8)	0 (0)	0 (0)	NS	0 (0)	0 (0)	0 (0)	1 (2.5)	NS
Transverse colon	0 (0)	0 (0)	1 (25.0)	0 (0)	0.041	0 (0)	0 (0)	0 (0)	1 (2.5)	NS
Unknown	2 (5.4)	1 (28)	0 (0)	1 (6.2)	NS	0 (0)	0 (0)	0 (0)	4 (10.0)	NS
Metastases					0.131					<0.001
No	15 (40.5)	10 (27.8)	1 (25.0)	9 (56.2)		7 (87.5)	7 (87.5)	21 (56.8)	0 (0)	
Yes	22 (59.5)	26 (72.2)	3 (75.0)	6 (37.5)		0 (0)	1 (12.5)	16 (43.2)	40 (100)	
Octreoscan					0.059					<0.001
Not Avid	15 (40.5)	10 (27.8)	1 (25.0)	9 (56.2)		3 (37.5)	4 (50.0)	25 (67.6)	3 (7.5)	
Avid	12 (32.4)	17 (47.2)	0 (0)	2 (12.5)		0 (0)	0 (0)	4 (10.8)	27 (67.5)	
NA	10 (27.0)	9 (25.0)	3 (75.0)	5 (31.2)		5 (62.5)	4 (50.0)	8 (21.6)	10 (25.0)	
Octreotide use					0.016					<0.001
No	24 (64.9)	14 (38.9)	4 (100)	10 (62.5)		8 (100)	8 (100)	28 (75.7)	8 (20.0)	
Yes	13 (35.1)	22 (61.1)	0 (0)	6 (37.5)		0 (0)	0 (0)	9 (24.3)	32 (80.0)	
LN involvement					0.610					<0.001
No	6 (16.2)	2 (5.6)	0 (0)	2 (12.5)		4 (50.0)	4 (50.0)	0 (0)	2 (5.0)	
Yes	23 (62.2)	30 (83.3)	3 (75.0)	11 (68.8)		0 (0)	0 (0)	35 (94.6)	32 (80.0)	
NA	8 (21.6)	4 (11.1)	1 (25.0)	3 (18.0)		4 (50.0)	4 (50.0)	2 (5.4)	6 (15.0)	
Vascular invasion					0.394					<0.001
No	8 (21.6)	7 (19.4)	1 (25.0)	3 (18.8)		7 (87.5)	3 (37.5)	5 (13.5)	4 (10.0)	
Yes	20 (54.1)	19 (52.8)	2 (50.0)	4 (25.0)		0 (0)	0 (0)	24 (64.9)	21 (52.5)	
NA	9 (24.3)	10 (27.8)	1 (25.0)	9 (56.2)		1 (12.5)	5 (62.5)	8 (21.6)	15 (37.5)	
Death					<0.001					0.018
No	34 (91.9)	28 (77.8)	0 (0)	10 (62.5)		7 (87.5)	5 (62.5)	34 (91.9)	26 (65.0)	
Yes	3 (8.1)	8 (36.0)	4 (100)	6 (37.5)		1 (12.5)	3 (37.5)	3 (8.1)	14 (35.0)	

Abbreviations: WHO, World Health Organization; AJCC/UICC, American Joint Committee on Cancer/Union Internationale Contre le Cancer; Dx, diagnosis; LN, lymph node; NA, not available; NS, non-significant.

Percentages reflect distribution among each grade or stage.

Presentation with appendicitis revealed association with both lower WHO 2010 grade and lower AJCC/UICC stages (p = 0.009 and p<0.001, respectively). Conversely, presentation with carcinoid symptoms was associated with higher AJCC/UICC stages (p<0.001) but not with WHO 2010 grading system. Appendix as the pathologic primary site showed association with both WHO 2010 G1 and AJCC/UICC stages I and II (p = 0.041 and p<0.001, respectively), while duodenum and transverse colon were associated with WHO 2010 G3 (p = 0.041) and ileum with AJCC/UICC stages III and IV (p = 0.001).

The presence of metastases and octreoscan avidity were both associated with higher AJCC/UICC stages (p<0.001 and p<0.001, respectively) but not with WHO 2010 grading [Table 2]. The presence of vascular invasion and lymph node involvement revealed positive association with AJCC/UICC staging (p<0.001 and p<0.001, respectively; Table 2).

### Survivals and Prognostic factors

Kaplan-Meier survival analyses revealed that median PFS for all patients was 104.0 months (95% CI, 85.8–122.1) with 5 and 10 year PFS rates of 83% and 51%, respectively. Median OS was 164.0 months (95% CI, 89.8–238.1) with 5 and 10 year OS rates of 75% and 34%, respectively. A total of 21 deaths were recorded including 15 deaths of iNETs-related causes.

**Figure 1:**
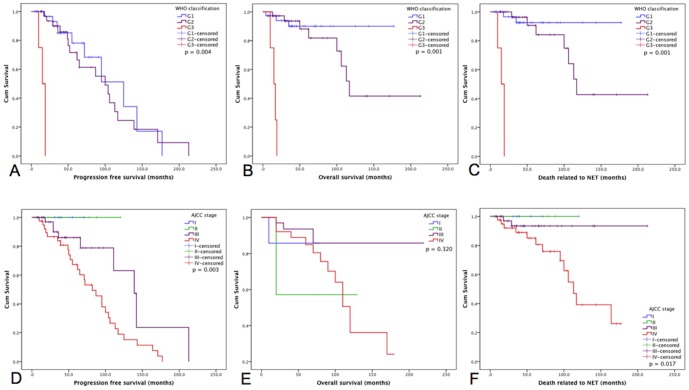
Kaplan-Meier survival analyses. (A) Progression free survival (PFS) according to WHO classification: Mean estimates were 107.6 months for G1, 99.8 months for G2 and 15.7 months for G3; N = 77 (p = 0.004). (B) Overall survival (OS) according to WHO classification: mean estimates were 161.4 months for G1, 140.6 months for G2 and 15.2 months for G3; N = 77 (p<0.001). (C) Disease-specific OS according to WHO classification: mean estimates were 165.8 months for G1, 144.2 months for G2 and 15.8 months for G3; N = 77 (p = 0.001). (D) PFS according to AJCC classification: median estimates were 70.0 months for stage I, 120.0 months for stage II, 138.4.0 months for stage III and 84.7 months for stage IV; N = 93 (p = 0.003). (E) OS according to AJCC classification: median estimates were 70.0 months for stage I, 120.0 months for stage II, 210.0 months for stage III and 110.4 months for stage IV; N = 93 (p = 0.320). (F) Disease-specific OS according to AJCC staging: median estimates were 70.0 months for stage I, 120.0 months for stage II, 210.0 months for stage III and 112.8 months for stage IV; N = 93 (p = 0.017).

Individual analyses for WHO 2010 classification showed an inverse association between grade and PFS (G1 107.6, G2 99.8 and G3 15.7 months, p = 0.004; Figure 1A) with 5 and 10 year PFS rates of 77% and 51% for G1 and 67% and 25% for G2, respectively; OS (G1 161.4, G2 140.6 and G3 15.2 months, p<0.001; Figure 1B) with 5 and 10 year OS rates of 90% for G1 and 82% and 41% for G2, respectively; and disease-specific OS (G1 165.8, G2 144.2 and G3 15.8, p = 0.001; Figure 1C) with 5 and 10 years disease-specific OS rates of 92% and 92% for G1 and 84% and 42% for G2, respectively; G3 tumors were not analyzable for survival rates.

Analyses for AJCC/UICC staging followed the same inverse association for PFS (stages I, II, III and IV with 70.0, 120.0, 138.4 and 84.7 months, respectively, p = 0.003; Figure 1D) with 5 year PFS rates of 100% for stage I, and 5 and 10 years PFS rates of 100% for stage II, 86% and 63% for stage III, and 64% and 19% for stage IV, respectively. No association was found with OS (stages I, II, III and IV with 70.0, 120.0, 210.0 and 110.4 months, respectively, p = 0.320; Figure 1E) with 5 year OS rates of 88%, 57%, 93% and 80% for AJCC/UICC stages I, II, III and IV, respectively whereas 10 year rates were 57%, 85% and 36% for stages II, III and IV. AJCC/UICC stage I patients did not reach a 10 year follow-up. Disease-specific OS (stages I, II, III and IV were 70.0, 120.0, 210.0 and 112.8 months, respectively, p = 0.017; Figure 1F) with 5 year disease-specific OS rates of 100%, 100%, 93% and 80% for AJCC/UICC stages I, II, III and IV, respectively. Corresponding 10 year rates were 100%, 93% and 39% for stages II, III and IV, respectively. No outcome differences were observed for age, gender or previous cancer history.

**Figure 2:**
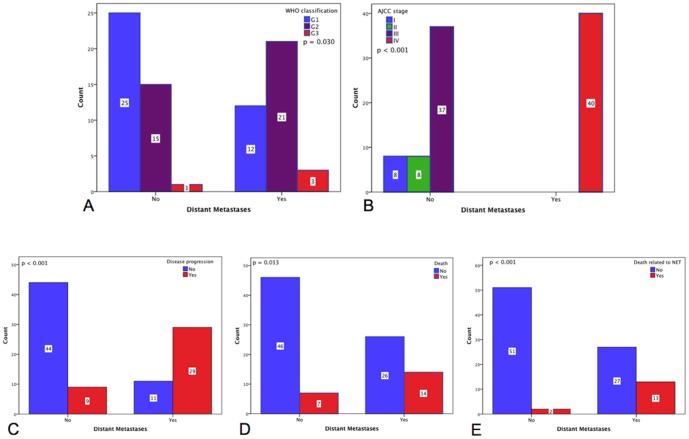
Presence of distant metastases according to WHO grading and AJCC staging. (A) WHO classification - The proportion of distant metastases was significantly different according to WHO grading as follows: 12 (32.4%) of G1 cases, 21 (58.3%) of G2 cases and 3 (75.0%) of G3 cases; N = 77 (p = 0.030); (B) Proportion of cases in each stage of AJCC classification - All the 40 patients (100%) with distant metastases were classified at stage IV; N = 93 (p<0.001); (C) Disease progression - Cases with distant metastases were associated to disease progression in 29 (72.5%) patients vs. 9 (17.0%) of patients without distant metastases; N = 93 (p<0.001); (D) Deaths occurred in 14 (35.0%) of patients with distant metastases vs. 7 (13.2%) of patients without distant metastases; N = 93 (p = 0.013); (E) Disease-specific deaths occurred in 13 (32.5%) of patients with distant metastases vs. 2 (3.8%) of patients without distant metastases; N = 93 (p<0.001).

**Figure 3:**
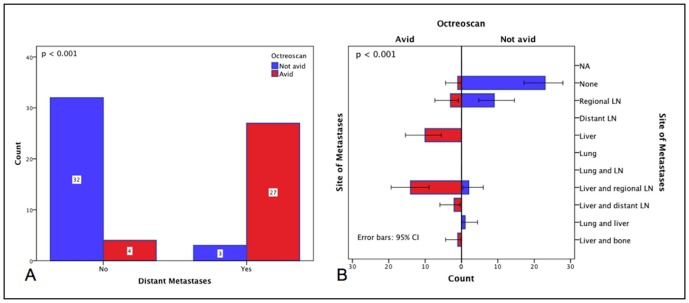
Octreoscan avidity and presence of distant metastases. (A) Cases with distant metastases presented with octreoscan avidity in 87.1% of patients vs. 12.9% of patients with non-distant metastases; N = 66 (p<0.001). (B) Octreoscan avidity and site of metastases. The likelihood of a positive octreoscan was associated with liver metastases [10/10 (100%)], liver and regional LN metastases [14/16 (87.5%)], liver and distant LN metastases [2/2 (100%)] and liver and bone metastases [1/1 (100%)]. Conversely, a negative octreoscan was associated with absence of metastases [23/24 (95.8%)] and regional LN involvement [9/12 (75.0%)]; Total N = 66, avid = 31 and not avid = 35; (p<0.001).

Larger primary tumors were positively associated with distant metastases (2.9±0.3 vs. 2.0±0.2 cm; p = 0.011), disease progression (3.0±0.3 vs. 2.0±0.2 cm; p = 0.001), death (4.3±0.8 vs. 2.2±0.2 cm; p = 0.013), and death related to iNETs (4.1±0.5 vs. 2.1±0.2 cm; p = 0.013). Distant metastases were proportionally associated with WHO 2010 grading with the following distribution: 32.4% (n = 12) of G1, 58.3% (n = 21) of G2 and 75.0% (n = 3) of G3 patients (p = 0.030; Figure 2A) and an association to AJCC/UICC stage IV (p<0.001; Figure 2B). The presence of distant metastases provided an increased risk of disease progression, death and death related to iNETs (p<0.001, p = 0.013 and p<0.001, respectively; Figures 2C,D,E) with an OR of 13.43 (95% CI, 4.90–36.85) and 3.14 (95% CI, 1.12–8.74) respectively, and a higher likelihood of showing avidity on octreoscanning (p<0.001; Figure 3A), especially in the group with liver metastases (p<0.001; Figure 3B).

### Somatostatin analog therapy

**Figure 4:**
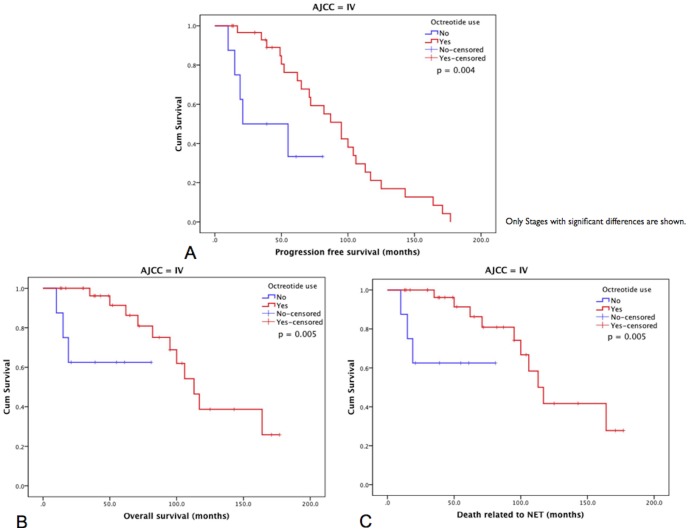
Kaplan-Meier survival analyses and octreotide use. (**A**) PFS median estimates for AJCC stage IV patients treated or not with octreotide were 91.2 and 50.0 months respectively; N = 40 (p = 0.004). (**B**) OS median estimates for AJCC stage IV patients treated or not with octreotide were 112.3 and 80.0 months respectively; N = 40 (p = 0.005). (**C**) Disease-specific OS median estimates for AJCC stage IV patients treated or not with octreotide were 114.5 and 80.0 months respectively; N = 40 (p = 0.013).

Octreotide LAR treatment was given to WHO 2010 G1 and G2 patients at AJCC/UICC stages III and IV (Table 2). The mean PFS among patients treated with octreotide LAR did not differ from that of patients who were not treated (97.9±8.9 vs. 107.2±9.7 months, respectively; p = 0.342). The same was true for OS (140.9±14.2 vs. 114.4±8.0, respectively; p = 0.115). Importantly, however, stratification by AJCC/UICC stage revealed statistical differences for PFS, OS and disease-specific OS between patients treated or not with octreotide LAR for stage IV (91.2 vs. 50.0 months, p = 0.004; 112.3 vs. 80.0 months, p = 0.005; and 114.5 vs. 80.0 months p = 0.005, respectively; Figure 4 and Figure S1).

We next performed a multivariate survival analysis, using a Cox proportional hazards regression model including WHO grade and AJCC stage. This analysis revealed a significant association of AJCC stage with PFS (p<0.001) with higher regression coefficients for the lowest grades. Octreotide use in this model, however, showed no significance for this parameter.

## Discussion

This study demonstrates that both the new WHO 2010 and AJCC/UICC classifications of iNETs provide clinically meaningful prognostic information. In our study cohort the most common presentation at diagnosis was abdominal pain (33.3%) and the most common primary site was the ileum (45.2%), in agreement with published literature [4], [6], [7]. We did not find differences in age or gender. Carcinoid symptoms were associated, as expected, with AJCC/UICC stage IV disease with liver metastases, but not with WHO 2010 grading. Presentation with appendicitis and an appendiceal primary were associated with lower WHO 2010 grade and AJCC/UICC stages, consistent with the more benign outcome of such tumors. Conversely, duodenal and proximal transverse colon primary sites were more frequently associated with WHO 2010 G3 status, reflecting more aggressive behavior. NETs arising from the ileum were more likely to be WHO 2010 G2, although not significant, and were associated with AJCC/UICC stages III and IV. Moreover, multiple primary small bowel lesions were noted in nearly 13% of patients, underscoring the need for comprehensive bowel visualization preoperatively. The presence of vascular invasion, as defined rigidly by the endocrine pathology group [21], which interestingly did not correlate with WHO 2010 grading, was statistically associated with advanced AJCC/UICC stages consistent with the underlying pathophysiologic mechanisms of metastatic disease.

The pathologic classification proposed by the WHO has evolved from purely morphologic [22] to one that also considers proliferative markers [1]. In our study cohort the new WHO 2010 grading system was statistically associated, in an inversely proportional manner, with different survival outcomes including PFS, OS and disease-specific OS. The same association was found for metastases to distant organs at diagnosis, reinforcing previous findings [23]–[25]. However, the WHO 2010 grading itself does not address other important clinical and imaging parameters relevant for patient management. In this context, the TNM staging proposed by AJCC/UICC was of prognostic value for PFS and disease-specific OS in our study population. It is noteworthy that the lower stages were less well represented in our cohort and had shorter follow-up periods compared with stages III and IV. This anticipated bias reflects the delay in diagnosis as such patients are typically asymptomatic. Nevertheless, our patients with stage I and II disease did not show recurrence or progression in contrast to the significantly diminished PFS rates over time in patients with stages III and IV disease. The same was observed for the OS rates when deaths related to iNETs were considered, reflecting the effectiveness of the AJCC/UICC staging system in providing prognostic survival outcomes. Ultimately, the presence of distant metastases, which was associated with AJCC/UICC stage IV and WHO 2010 G2 and G3, increased the risk of disease progression and death. Nonetheless, due to the small number of G3 tumors in our cohort the data from these cases must be interpreted with caution.

Serum chromogranin A and urinary 5-HIAA are commonly used as clinical biomarkers of disease activity. However as shown in the current study, these measurements are considerably less sensitive than tissue expression of chromogranin A and serotonin. Such discrepancies can be accounted for by technical differences in assay performance. It is also well documented that peptide synthesis may differ from secretion; resulting in storage that can be detected by tissue studies but not measured in the circulation. Moreover, it is increasingly evident from targeted therapeutic trials that systemic peptide secretion can be frequently uncoupled from endocrine tumor progression [26]. Thus, until more sensitive biomarkers are available, imaging studies will remain pivotal in the longitudinal follow up of patients with iNETs.

Octreoscanning, available in 71% of our patients, revealed that avidity is statistically associated with the presence of distant metastases and therefore AJCC/UICC stage IV. The majority of cases with avidity had liver involvement. No association was found with WHO 2010 grade in this group of patients. Nevertheless, this examination is known to have a low sensitivity but high specificity for NETs [27]. Our results, together with previous evidence that octreoscan avid NETs have better prognosis when compared with non-avid disease [28], are consistent with the potential prognostic value of octreoscanning in newly diagnosed patients with iNETS.

The use of octreotide LAR improved outcomes for AJCC/UICC stage IV patients, increasing PFS, OS, and disease-specific OS advantages. Before publication of the PROMID study, octreotide LAR was mainly indicated for symptoms related to the carcinoid syndrome, although many series had shown stability and sometimes regression of NETs in patients in response to this drug [13]. This drew attention to the antiproliferative effects of octreotide, which were later validated *in vitro* [29]. It is clear that our results could be biased by the fact that this is a retrospective study and not a clinical trial. Our finding of a longer time to progression among patients treated with octreotide LAR is consistent with the PROMID [13] and other long-term retrospective studies [30], [31]. Although we noted no correlation with WHO 2010 grade, it is appreciated that G3 NETs are less likely to be considered for octreotide LAR treatment, and require more aggressive approaches [15]. Therefore, our results are in agreement with the recommendation of treatment with somatostatin analogs for patients with G1 and G2 grade iNETs.

In summary, we conclude that both the WHO 2010 grading and the AJCC/UICC staging systems are useful predictors of iNETs behavior. While the AJCC/UICC staging is more suitable for treatment decisions regarding octreotide therapy, the two systems provide complementary information and should be used in concert in the management of this disease.

## Supporting Information

Figure S1 Kaplan-Meier survival analyses and octreotide use. (A) PFS mean estimates for WHO G1 patients treated or not with octreotide were 106.6 and 78.2 months respectively; N = 37 (NS). (B) PFS mean estimates for WHO G2 patients treated or not with octreotide were 90.7 and 129.0 months, respectively; N = 36 (NS). (C) OS mean estimates for WHO G1 patients treated or not with octreotide were 164.0 and 83.2 months respectively; N = 37 (NS). (D) OS mean estimates for patients with G2 tumors treated or not with octreotide were 133.0 and 131.8 months respectively; N = 36 (NS). (E) PFS median estimates for AJCC stage III patients treated or not with octreotide were 140.7 and 117.5 months respectively; N = 37 (NS). (F) OS median estimates for AJCC stage III patients treated or not with octreotide were 210.0 and 140.0 months respectively; N = 37 (NS). All other grades and stages that received octreotide treatment showed no significant differences.
(TIFF)
