# Supplementary figures and images for: Evaluation of the WHO 2010 Grading and AJCC/UICC Staging Systems in Prognostic Behavior of Intestinal Neuroendocrine Tumors
Source: PLoS One. 2013 Apr 19;8(4):e61538. doi: 10.1371/journal.pone.0061538 (PMC3631242; doi:10.1371/journal.pone.0061538)

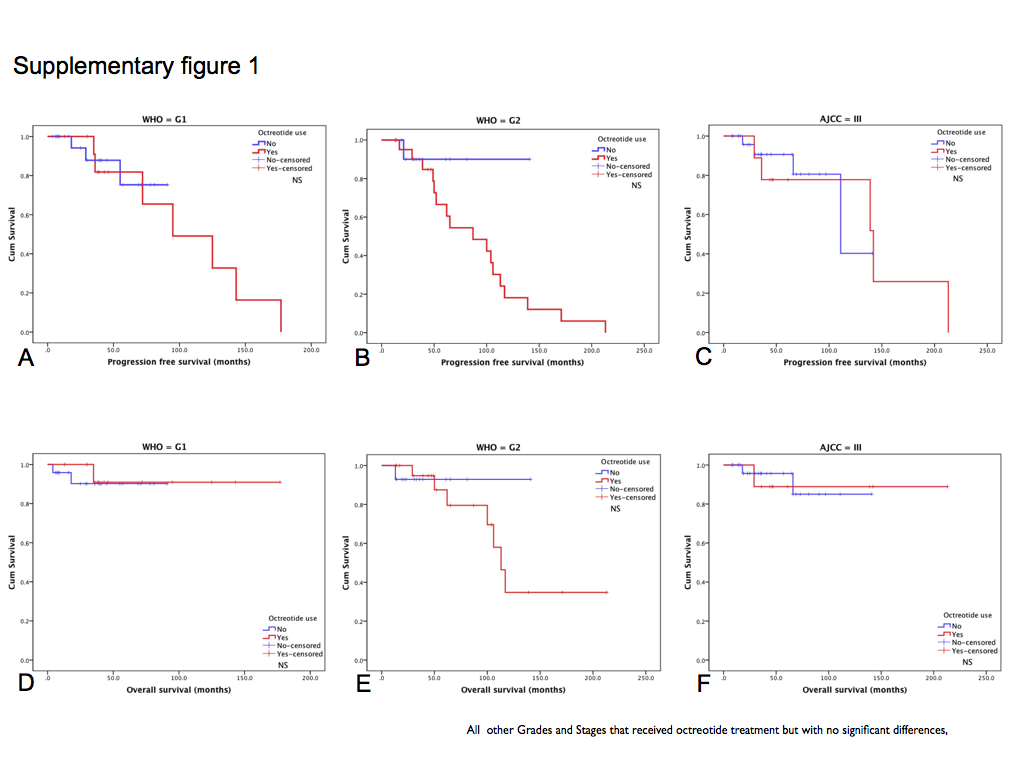

Supplement: Figure S1 — Kaplan-Meier survival analyses and octreotide use. (A) PFS mean estimates for WHO G1 patients treated or not with octreotide were 106.6 and 78.2 months respectively; N = 37 (NS). (B) PFS mean estimates for WHO G2 patients treated or not with octreotide were 90.7 and 129.0 months, respectively; N = 36 (NS). (C) OS mean estimates for WHO G1 patients treated or not with octreotide were 164.0 and 83.2 months respectively; N = 37 (NS). (D) OS mean estimates for patients with G2 tumors treated or not with octreotide were 133.0 and 131.8 months respectively; N = 36 (NS). (E) PFS median estimates for AJCC stage III patients treated or not with octreotide were 140.7 and 117.5 months respectively; N = 37 (NS). (F) OS median estimates for AJCC stage III patients treated or not with octreotide were 210.0 and 140.0 months respectively; N = 37 (NS). All other grades and stages that received octreotide treatment showed no significant differences. (TIFF) [file pone.0061538.s001.tiff]
